# Water-soluble carbohydrates of root components and activity rhythms at vegetative growth stage of *Artemisia scoparia* in northeastern grassland of China

**DOI:** 10.1371/journal.pone.0176667

**Published:** 2017-05-09

**Authors:** Shiyu Wang, Yunfei Yang, Heng Zhi

**Affiliations:** Key Laboratory of Vegetation Ecology, Ministry of Education, Institute of Grassland Science, Northeast Normal University, Changchun, Jilin province, P.R. China; The University of Eastern Piedmont, ITALY

## Abstract

The root system of perennials is composed of the roots of different growth years. The nutrient storage capacities and activities of roots are an important basis for judging root components and plant senescence. In this research, changes in the contents of water-soluble carbohydrate (WSC) were used as indicators of the nutrient storage and activity of roots of different life years. From the early resprouting stage to the rapid growth stage, *Artemisia scoparia* L. plants of 1–3 age classes were sampled and measured once every 18 days. The nutrient storage capacities and activity rhythms of plant root components of the three age classes were analysed quantitatively. Among the *A*. *scoparia* population in northeast China, the nutrient storage capacities of 1a/2a plant root collars and 2-year old roots were generally large, whereas those of 3a plant root collars and 3-year old roots were significantly reduced. As for changes in the WSC content in the root system at the 18 day resprouting stage, the decline rates in the root collars of the 1a and 2a plants were 102 and 109 times those of the 3a plants, respectively. The decline rate in the 2-year old roots of the 1a plants was 1.8 times that of the 2a plants and 29.6 times that of the 3a plants. When nutrients were most active, all root components of the 1a plants entered into the resprouting stage, but the 2/3-year old roots of the 2a plants lagged behind. All the root components of the 3a plants generally lagged. At the vegetative growth stage, the WSC contents in all root components of the 1a plants declined logarithmically. For the 3a plants, the content in the root collars decreased linearly with that in the 3-year old roots. The older root components (3-year old roots) of the 2a plants and all root components of the 3a plants exhibited signs of aging.

## Introduction

Roots are important organs for plants to absorb moisture and mineral nutrients, and they also function as vital nutrient storage organs for perennials to achieve overwintering regeneration [1, 2]. For all perennial plants in temperate grassland, the underground organs are perennial, whereas the aboveground parts always change in a regular pattern of resprouting in spring, vigorously growing in summer, withering and yellowing after fruiting in autumn and suffering whole-plant death in winter; such rhythmic variation leads to the regulation of physiological metabolism with nutrients separately transported to the underground and aboveground parts [3].

Water-soluble carbohydrate (WSC) are an important active substances in plant physiological metabolism [4, 5], and its content varies with plant species [6, 7], ecotypes [8–11]and organs [12]. Even in the same plant species, the content also varies with defoliation [13, 14], ecological stress [15, 16] and habitat [17]. At the end of the growth season of temperate grassland plants, the regenerative underground organs and perennial basal parts of stems (the rhizomes, root collars or tillering nodes) occupy a considerable proportion of the total biomass (generally 3–5 times as the aboveground biomass), and the storage of nutrients is mainly based on carbohydrates [18]. Numerous studies have shown that carbohydrates in storage organs play a crucial role in plant growth and development. For almost all of the grasses, germination and resprouting at the beginning of the growing season consume high levels of carbohydrates stored in their underground organs [19–21]. A large amount of carbohydrates are accumulated at the end of the growing season after fruiting [1, 8, 9]. During growth and development, the contents of carbohydrates vary with biological behaviours of plant species, such as growth rate or phenological phase [1, 19, 20, 22]. It would be meaningful for the role of photosynthesis on carbohydrate storage [23, 24], since this occurs due to a favourable balance between the input by synthesis and the use by growth and maintaining metabolism [25]. However, given the difficult in identifying the age of underground parts of perennial herbaceous plants, reports on the WSC contents, nutrient storage and activity, aging process or any other physiological metabolic information in different storage organs of the same species are few [26]. For the research which was the difference and the change rules of soluble carbohydrate content in the root storage components of different life years in the spring nutrient consumption phase has not yet been reported.

*Artemisia scoparia* is a member of degraded grassland plant communities in the Songnen Plain, and it is a pioneer species in the succession of plant communities of abandoned farmland [21]. *A*. *scoparia* is widely distributed in temperate regions of China, and on the basis of the Flora of China, its individual life history is diverse, such as annuals, biennials and perennials [27]. According to our research on the life forms and life history characteristics of grassland plants found that the *A*. *scoparia* population in frequently disturbed habitats is composed of 1-year, 2-year and more than 3 -year older plant individuals. Most of those in grazing habitats are perennial. Previous studies on *A*. *scoparia* reported seed weight and germination characteristics [28, 29], seedling survival [30], root growth [31], biomass distribution [32], leaf morphology and anatomy [33, 34], gender expression and reproductive output [35], intraspecific and interspecific competitions [36], allelopathy [37, 38] and gene expression in the biological synthesis of artemisinin [39]. The root system of *A*. *scoparia* is a taproot type, and grows many new lateral roots from the taproot every year. Every individual varies with settling time, and its root system is composed of roots at different ages. Therefore, differences among the root components of the *A*. *scoparia* population plant individuals of different age classes in the WSC content, and the changing rate at the vegetative growth stage, were studied. Such differences were associated with material storage, activity and aging. Their activity rhythms were modelled and quantitatively characterised. The significance of the above items is in the new ideas and methods of investigating the root system of perennial herbaceous plants. The WSC content of different age class was broadened, providing a theoretical basis for a thorough understanding of the life plasticity of *A*. *scoparia* individuals and the physiological changes in root senescence.

## Material and methods

### Study area

Samples were collected from a fenced meadow of degraded *Leymus chinensis* (123°45′-123°47′E, 44°40′-44°44′N) at the Songnen Grassland Ecological Research Station of Northeast Normal University in the Stud Farm of Changling County, Jilin Province, in the southern Songnen Plain. There were no specific permissions required for the study area as our institute were authorized to run and manage the research station. The *A*. *scoparia* population is diverse and well distributed. The early growth stage in which the plot demonstrates community succession with low coverage was set as the sampling site with an area of 600 m^2^. In the plot, *L*. *chinensis*, *Hierochloe glabra*, *Kalimeris integrifolia*, *Arundinella hirta*, *Calamagrostis epigeios*, *Carex duriuscula* and other perennial clonal plants were distributed in a small patchy pattern and mixed with numerous annual plants, such as *Setaria viridis*, *Chloris virgata*, *Digitaria chrysoblephara* and *Salsola collina*. The soil is alkalised meadow soil [40]. The investigated plot is located in a semi-arid and semi-humid temperate zone with typical continental monsoon climate characteristics: windy and drought in spring; warm, wet and rainy in summer; temperate and moderate rainfall in autumn; and cold, windy and drought in winter. The annual average temperature is 4.6°C–6.4°C, with the mean minimal is -20.6°C in January and the mean maximal is 28.2°C in July. The frost-free season lasts 120–140 days. The average annual rainfall is 400–500 mm, and it is mainly concentrated from June to August; the annual evaporation is 1200–1400 mm, which is about two to three times as the annual rainfall [41, 42].

### Study species

It should be confirmed that the field studies did not involve endangered or protected species. Upon observing a large number of samples for preliminary experiment, results showed that the taproot of *A*. *scoparia* is well developed and the lateral roots are clear. In the frequently disturbed and degraded grassland of the Songnen Plain, 1-3-year old plants are ubiquitous in the *A*. *scoparia* population, and individuals that are at least 2 years old develop new thick lateral roots in the middle and late growth season every year. For plants of different age classes, the roots of the year are fleshy and white. With the growth and development of the aboveground aerial parts after resprouting in the following year, the fleshy roots are gradually lignified, turning from white to pale yellow and to deep yellow. The degree of lignification increases with the years of life, and the colour also deepens. Thus, obvious differences exist between the roots formed in different years in the colour, but the root systems of the plants of different age classes have components of the same year (S1 Fig).

### Sampling method

From the early resprouting stage to the vegetative growth of the rapid growth stage, *A*. *scoparia* plants of 1–3 years old were sampled once every 18 days for a total of five times. Specifically, the sampling dates were April 10, April 27, May 16, June 3 and June 21.

When sampling, first, all plant samples were with aerial parts of living individuals, and then ages from the dead branches on the root collars were preliminarily assessed. The aboveground and underground parts of the plants were dug out, and all the branches remained in a natural state. The sampled plants were packed into plastic bags and labelled as the age classes that were preliminarily identified. Twenty plant samples were taken for every age class and quickly sent to the laboratory for fixing for 15 min at 105°C.

### Identifying the age of different root components

The root systems of the plants of different age classes for fixing for 15 min at 105°C were divided into root collars, taproots and lateral roots of different years of life. The ages of the *A*. *scoparia* plants were identified by a technique that had been previously adopted for the age structure of the *Compositae* population [43, 44]and based on the regenerative and secondary roots. The lateral roots were measured by a combination of colour, lignification and years of life. The root collars and lateral roots of different age classes were cut and separated, labelled with unique ordinal numbers, once again placed in an oven at 80°C and dried to their respective constant weights. They were crushed, sieved with a 40-mesh screen and stored in a desiccator.

### Determining the WSC

In each sample accurately weighing 0.05g, added 20 ml deionized water in the glass tube for 30 min, and then heated to boiling water (100°C) 1h, then take it after extraction of the sample of the centrifugal clear liquid was used to measure analysis. Each sample repeated three times. Under A 96-well, flat-bottomed, polystyrene microplate reader (Titertek Multiskan Ascent, Model No:354, Germany), and the absorbance recorded at a wavelength of 490 nm, phenol-sulfuric colorimetry was adopted for determining the WSC contents in the *A*. *scoparia* root components of different age classes plants [45, 46]. The root components of the plants of different age classes were repeatedly determined for four samples. The WSC contents in the samples were calculated using the following Formula (1):

$$C=\frac{SC\times SV\times SDM}{SM\times 10^{3}}\times 100\%$$ (1)

Where *C* is the WSC content (%), *SC* is the sample concentration (mg/ml), *SV* is used to measure solution volume (mL) of the samples, *SDM* is sample dilution multiple, and *SM* is sample mass (g).

### Statistical analysis

The WSC contents in the root collars, as well as the two- and three-year roots, of *A*. *scoparia* were compared via ANOVA and Duncan’s multiple-range test based on the same components among the plants of three age classes at the same stage. The average WSC contents of the same root components of different age class plants were compared by ANOVA and Duncan’s multiple-range test based on the different components at the same time. The change rate in the WSC content in the root components of the plant of different age classes at different stages of vegetative growth is calculated by the following Formula (2):

$$R=\frac{C_{\text{t}+1}-C_{t}}{D_{i}}$$ (2)

where *R* represents the change rate (%, d^-1^), *C* represents the WSC content and *D*_*i*_ represents the sampling time *t*+1 and interval (days) *t*. Five times in total sampling time was 73 days, as calculated from April 10, the first time sampling on the start date of growth until June 21, the last time sampling. Using the linear, logarithmic and exponential functions, regression analysis and significance test were performed for the measured WSC contents (n = 4×5) in the root components of the plants of the three age classes and their respective averages of different age class (n = 3×5), average of all of the components (n = 5) and the growth time. SPSS17.0 statistical software package was used for all statistical analyses. All statistical analyses were performed with SPSS 17.0 statistical package.

## Results

### Differences of the WSC contents among the root components and different year old roots

**Fig 1:**
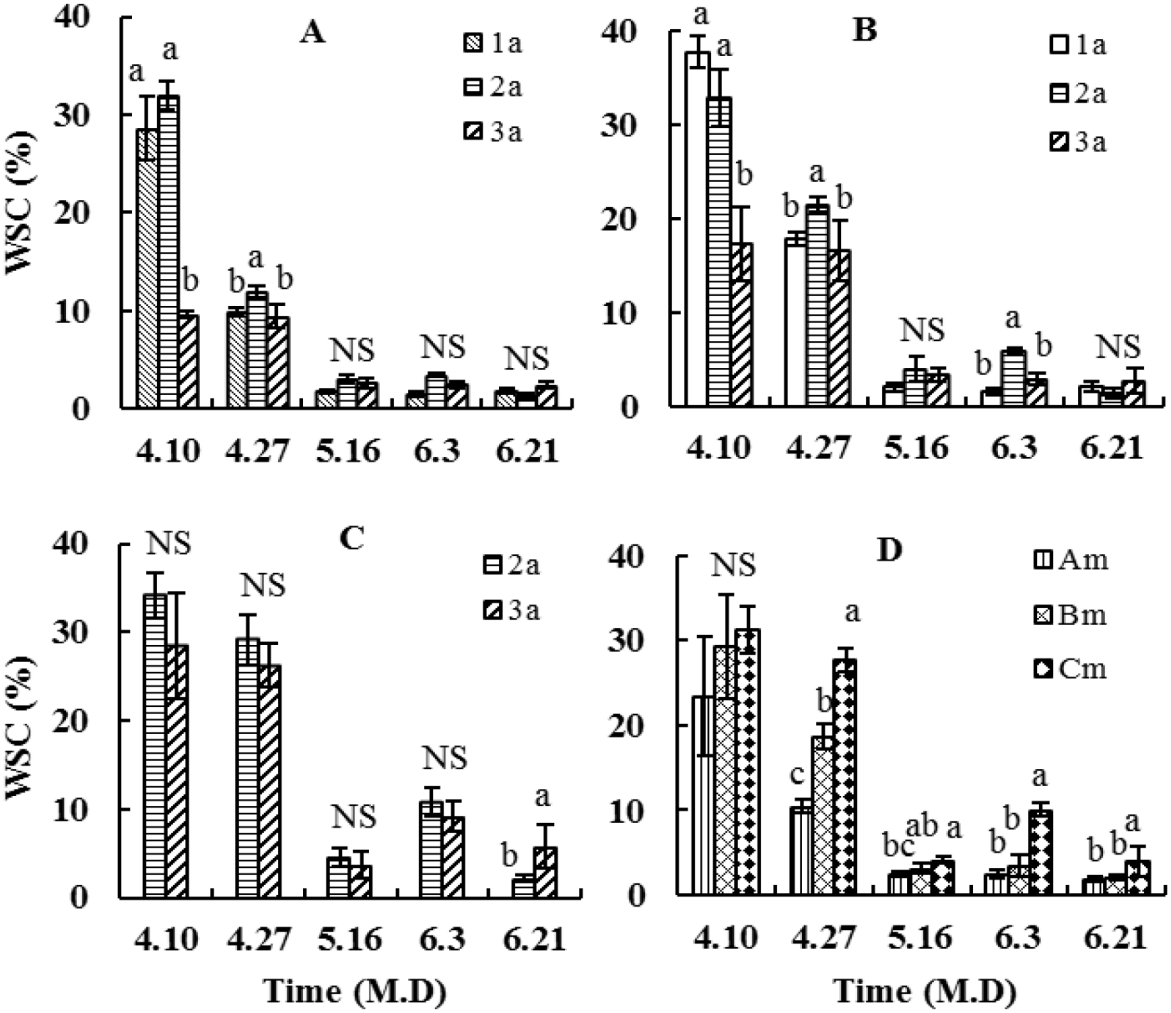
Comparison among the *Artemisia scoparia* plants of three age classes in the contents of water-soluble carbohydrate (WSC) in the root components at different times. (A, root collars; B, 2-year old root; C, 3-year old root; D, average of same root components of the plants of three age class; In icon from A to C, the letter “a” is age class of the plants which is 1a, 2a, 3a; above the data column(mean±SE), different small letters mean significant difference(p<0.05), NS is no significant difference(p>0.05) in from A to C among three age classes; Am, average of root collars, Bm, average of 2-year old roots, Cm, average of 3-year old roots in D icon; the small letters or NS above the data column(mean±SE) are the same as the meaning from A to C in D among the root components).

At the vegetative growth stage of *A*. *scoparia* in grasslands of northeast China, the WSC content in the root collars of the 2a plants in early April of the early resprouting stage was the highest, whereas that of the 3a plants was the lowest, thereinto, the 3a plants was significantly lower(P<0.05) than the 1a or 2a plants. In late April, the 2a plants was significantly higher than the 1a and 3a plants; entering into the vigorously growing stage in mid-May, insignificant differences (P>0.05) were found among the plants of the three age classes (Fig 1A). The WSC content in the 2-year old roots of the 1a plants at the early resprouting stage in early April was the highest, whereas that of the 3a plants was the lowest, the 3a plants was significantly lower than the 1a and 2a plants, in mid-May and late June, no significant differences were observed among the plants of the three age classes (Fig 1B). In terms of the WSC content in the 3-year old root from the early resprouting stage in early April to the vigorously growing stage in early June, the difference was not significant between the 2a and the 3a plants, furthermore, the 3a plants was significantly higher than the 2a plants in late June (Fig 1C). These findings reflected the differences among *A*. *scoparia* plants of different age classes in the nutrient storage ability and the activity of the same root components.

In terms of the WSC content in the root components, upon comparing the average of the three age class plants (Fig 1D) at the vegetative growth stage, the 3-year old roots was the highest, whereas the root collars was the lowest. Although the WSC content differences among the three root components at the early resprouting stage in early April were not significant, the 3-year old roots at any other stage was significantly higher than the root collars. The WSC content in the 2-year old roots was significantly higher than the root collars only in late April, and both were at the same level at other stages. These results reflected the differences among the root components of the *A*. *scoparia* population in nutrient storage capacity and the activity. Differences among the root components in activity at different stages were better reflected using the following quantitative indicators.

### Changes in the WSC contents in root components at different stages of vegetative growth

During the vegetative growth stage of *A*. *scoparia* in grasslands in northeast China, the change rate in the WSC content in the root component of the plants of different age classes at different stages of vegetative growth fluctuated irregularly (Table 1). The minus value indicates the daily decline rate, whereas the plus value indicates the daily increase rate. As Table 1 shows, the change rate in the WSC content in the root components of the plants of different age classes before mid-May was a negative value, but the absolute value was generally relatively large. After mid-May, a positive value appeared, but the absolute value was relatively small. In terms of the WSC content in the root collars within 18 days of the resprouting stage, the decline rate of the 2a plants was the highest (−1.1755%·d^-1^) among the plants of the three age classes, but the 1a plants (−1.1026%·d^-1^) was roughly the same level as the 2a plants. The decline rates of the 1a and 2a plants were 101-fold and 108-fold, respectively, higher than that of the 3a plants. From the 2-year roots, the decline rate of the 1a plants was the highest (−1.1733%·d^-1^) and 1.8-fold and 28.6-fold compared with those of the 2a and 3a plants, respectively. From the root collars and 2-year old root within 19–37 days, the decline rate of the 2a plants was the highest, but the plants of the three age classes slightly changed. The coefficients of variations of the root collars and 2-year old roots were 13.8% and 14.1%, respectively.

**Table 1:** Change rate in the WSC content in the *Artemisia scoparia* root components of plants of three age classes at different stages of vegetative growth.

Root component	Age class of plants	Growth time range (day, %·d^-1^)			
		1–18	19–37	38–55	56–73
**Root crown**	1	-1.1026	-0.4230	-0.0187	0.0214
	2	-1.1755	-0.4722	0.0245	-0.1163
	3	-0.0108	-0.3575	-0.0098	-0.0019
	Mean	-0.7630	-0.4176	-0.0013	-0.0322
	SD	0.6524	0.0576	0.0228	0.0737
**Root of 2-year old**	1	-1.1733	-0.8303	-0.0251	0.0277
	2	-0.6663	-0.9216	0.1086	-0.2514
	3	-0.0396	-0.6940	-0.0284	-0.0102
	Mean	-0.6264	-0.8153	0.0184	-0.0779
	SD	0.6264	0.8153	0.0184	0.0780
**Root of 3-year old**	2	-0.2888	-1.2939	0.3491	-0.4784
	3	-0.1372	-1.1853	0.3079	-0.1906
	Mean	-0.2130	-1.2396	0.3285	-0.3345
	SD	0.1072	0.0767	0.0292	0.2035

As shown at the growth stage in Table 1, in the root collars of the 1a and 2a plants, and 2-year old roots of the 1a plants the decline rate within the first 18 days at the early growth stage was the highest; From the 2-year and 3-year old roots of the 2a plants, the decline rate within 19–37 days was the highest. From the root collars and the 2-year and 3-year old roots of the 3a plants, the decline rate within 19–37 days was the highest. Within 38–55 days, the root collars, the 2-year and 3-year old roots of the 2a plants, and the 3-year old roots of the 3a plants increased to varying degrees, but nutrient accumulation was not stable. Moreover, a decrease in nutrient consumption was found in the next 56–73 days. The root collars and 2-year old roots of the 1a plants within 56–73 days were more or less to increase nutrient accumulation, but the 2-year old roots of the 3a plants showed a reduction in nutrient consumption.

### Rhythmic variation in the WSC content in the root component of plants of different age classes with growth times

Regression analysis and significance test (Table 2) were conducted in samples from grasslands of northeast China, from the early resprouting stage in early April to the vigorously vegetative growth period in late June. The regression equations for changes in the WSC contents in the root components of the *A*. *scoparia* plants of the three age classes reached extremely significant level with the growth time(days) (p < 0.01). Based on the coefficient of determination (R^2^), the logarithmic functions of the root collars and 2-year old roots of the 1a and 2a plants were the highest, the linear functions of the 3-year old roots of the 2a plants and the root collars and 3-year old roots of the 3a plants were the highest and the exponential function of the 2-year old roots of the 3a plants was the highest. According to the change rules revealed by the best-fit equation with the highest coefficient of determination, the WSC contents in all root components of the 1a plants declined logarithmically during the vegetative growth stages of the *A*. *scoparia* population. The 2-year old roots of the 3a plants declined exponentially, whereas the other two components decreased linearly. The root collars and 2-year old roots of the 2a plants declined logarithmically as the root components of the 1a plants, and the 3-year old roots of the 2a plants also logarithmically decreased with the 3-year old roots of the 3a plants.

**Table 2:** Regression equation parameters and significance test of the WSC content (y, %) in the *Artemisia scoparia* root components of plants of three age classes and the growth times (x, day).

Component	Age class of plant	n	Equation	Parameter		R^2^	*P*
				a	b		
**Root crown**	1	20	Ln	28.379	-6.6444	0.9226(1)	<0.01
			E	18.859	-0.0410	0.7745(2)	<0.01
			L	21.226	-0.3400	0.6566(3)	<0.01
	2	20	Ln	31.969	-7.3039	0.9726(1)	<0.01
			E	26.105	-0.0426	0.8831(2)	<0.01
			L	24.469	-0.3836	0.7292(3)	<0.01
	3	20	L	9.6604	-0.1185	0.7049(1)	<0.01
			E	9.694	-0.0232	0.6856(2)	<0.01
			LN	10.598	-1.7899	0.5920(3)	<0.01
	Mean	15	E	16.952	-0.0351	0.7807(1)	<0.01
			Ln	23.649	-5.2460	0.7535(2)	<0.01
			L	18.452	-0.2807	0.5863(3)	<0.01
**Root of 2-year old**	1	20	Ln	38.585	-8.8940	0.9397(1)	<0.01
			E	28.832	-0.0463	0.7615(2)	<0.01
			L	30.027	-0.4828	0.7525(3)	<0.01
	2	20	Ln	34.696	-7.2936	0.8376(1)	<0.01
			L	29.045	-0.4330	0.8024(2)	<0.01
			E	35.877	-0.0451	0.7709(3)	<0.01
	3	20	E	18.641	-0.0356	0.6299(1)	<0.01
			L	17.313	-0.2376	0.5816(2)	<0.01
			Ln	19.223	-3.5991	0.4911(3)	<0.01
	Mean	15	E	26.374	-0.0393	0.7961(1)	<0.01
			Ln	30.835	-6.5956	0.7772(2)	<0.01
			L	25.462	-0.3845	0.7177(3)	<0.01
**Root of 3-year old**	2	20	L	32.863	-0.4541	0.7511(1)	<0.01
			E	37.158	-0.0361	0.7092(2)	<0.01
			Ln	37.146	-7.0924	0.6743(3)	<0.01
	3	20	L	27.439	-0.3466	0.5518(1)	<0.01
			Ln	31.009	-5.5159	0.5142(2)	<0.01
			E	24.657	-0.0264	0.3974(3)	<0.01
	Mean	10	L	30.151	-0.4003	0.7474(1)	<0.01
			Ln	34.078	-6.3042	0.6820(2)	<0.01
			E	31.178	-0.0296	0.6507(3)	<0.01
**Total**	Mean	5	Ln	28.951	-6.0166	0.8825(1)	<0.05
			E	24.836	-0.0340	0.8201(2)	<0.05
			L	24.005	-0.3495	0.8094(3)	<0.05

Note: Ln, atural logarithmic equation; L, linear equation; E, exponential equation. The number is order of three suitable equations in parentheses in R^2^ column.

In all the equation parameters fitted in Table 2, a represents the intercept. A negatively correlated equation yields the theoretical maximum or the starting point of the fitted curve downward, whereas and a positively correlated equation yields the theoretical minimum or the starting point of the fitted curve upward. ‘b’ represents the change rate. The ‘positive’ or ‘negative’ sign only indicates the direction of the curve change, and the absolute value is shown only for comparison. In the comparison of the equation parameters, only equations of the same law were comparable. The biological significance of ‘a’ was the theoretical nutrient storage.

**Fig 2:**
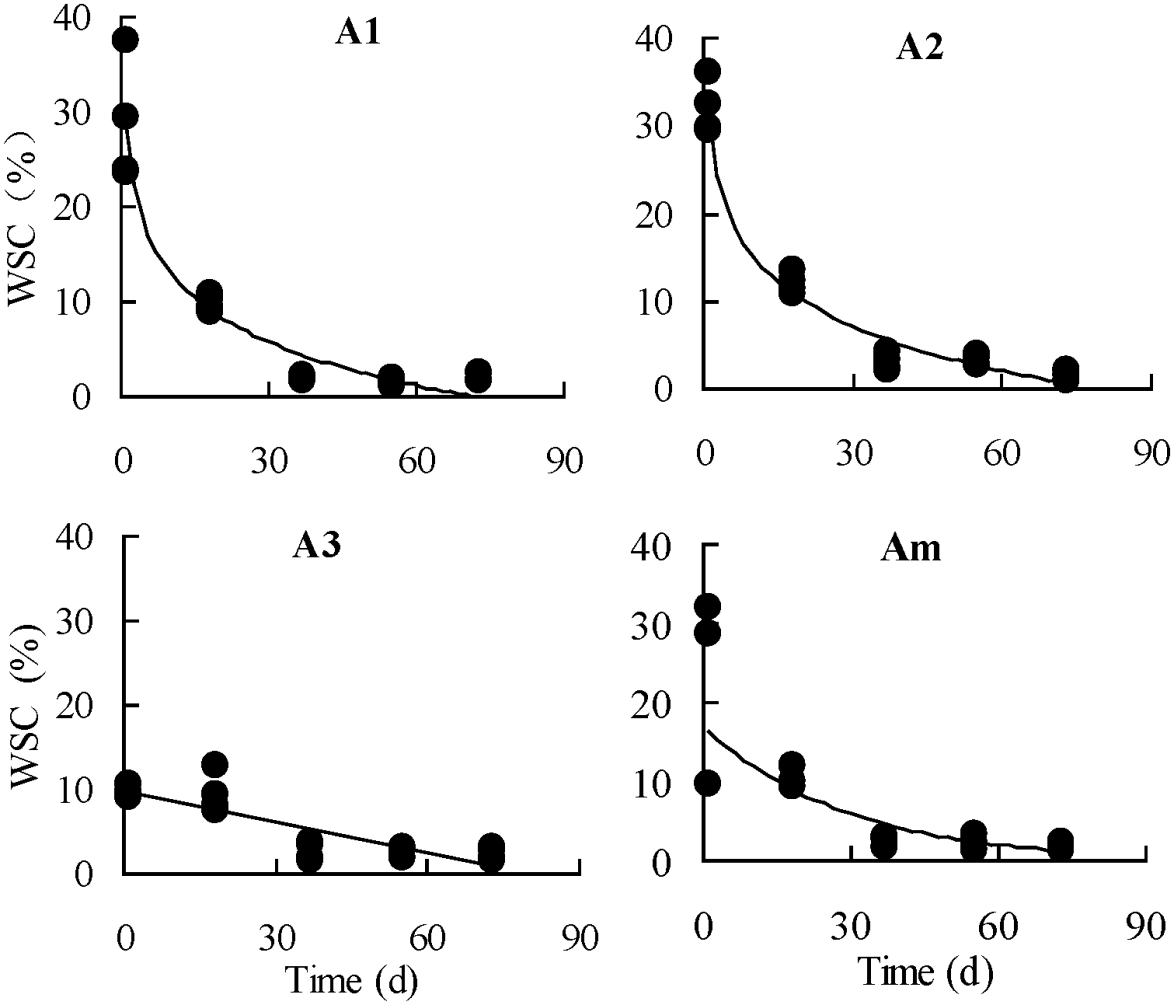
Observed value and best-fit equation curves of seasonal changes in the contents of water-soluble carbohydrate (WSC) in the root collars of *Artemisia scoparia* plants of three age classes. (A1, 1a plants; A2, 2a plants; A3, 3a plants; Am, average of three age classes).

**Fig 3:**
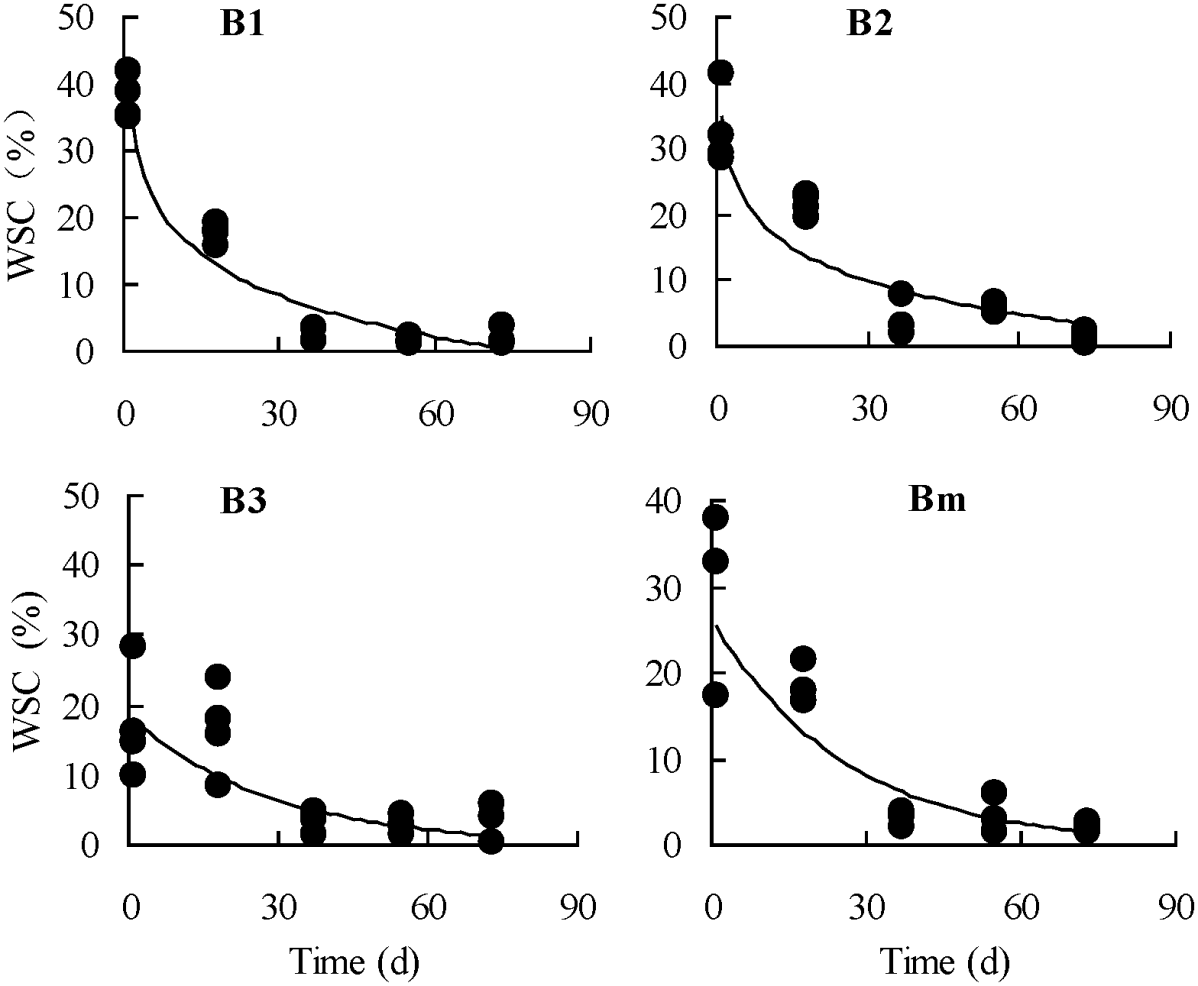
Observed values and the logarithmic fitting curves of seasonal changes in the contents of water-soluble carbohydrate(WSC) in the 2-year old roots of *Artemisia scoparia* plants of three age classes. (B1, 1a plants; B2, 2a plants; B3, 3a plants; Bm, average of the plants of three age classes).

**Fig 4:**
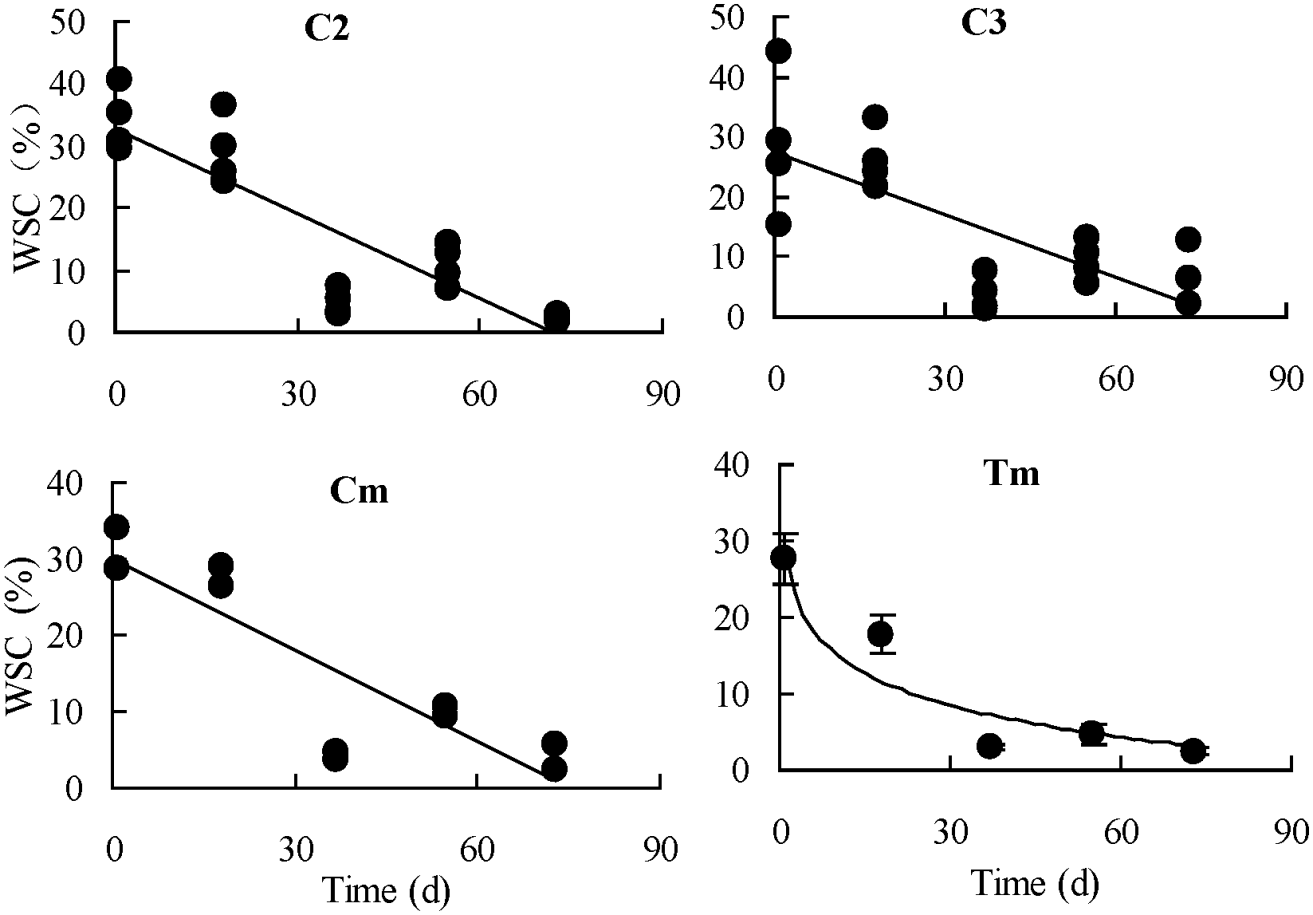
Observed values and the logarithmic fitting curves of seasonal changes in the contents of water-soluble carbohydrate (WSC) in the 3-year old roots of 2a and 3a plants of *Artemisia scoparia*, and the average of total root components. (C2, 2a plants; C3, 3a plants; Cm, average of 2a and 3a plants; Tm, average of total root components as root collars, 2-yaer old roots and 3-year old roots of the plants of three age classes).

As shown in Table 2, in the root components of the 1a plants of the *A*. *scoparia* population, the absolute values of the natural logarithmic equation parameters (a and b) of the 2-year old roots were greater than those of the root collars. In particular, ‘a’ increased by 36% and b increased by 33.9%, indicating that nutrient storage and activity of the 2-year old roots of the 1a plants at the vegetative growth stage were greater than those of the root collars. For the 2a plants, the 2-year old roots was also greater than the root collars, but a only increased by 8.5% and b did substantially the same, indicating that nutrient storage of the 2-year old roots increased compared with the root collars, but the activity was the same. In the root components of the 3a plants, the absolute values of the linear equation parameters (a and b) of the 3-year roots were the highest, followed by those of the 2-year old roots, whereas those of the root collars were the lowest. These findings indicated that the 3-year old roots in the root components of the 3a plants still had relatively higher storage and activity. However, compared with the 2a plants, the storage and activity in different root components of the 3a plants decreased to varying degrees. From the early resprouting stage in early April to the vigorously vegetative growth period in late June, the observed values of the WSC content in the root components the plants of different age classes at different growth stages and the best-fit curve of its variation with time are shown as Figs 2–4.

## Discussion

### WSC content variation and aging process of the *A*. *scoparia* root components

The WSC contents in different root components and their variation are important indicators for plant nutrient storage capacity and the physiological metabolism activity [14, 47]. In the temperate steppe zone, nutrient storage capacity of the root system of perennial herbaceous plant is the largest generally before dormancy [26]. According to the present research results in relative terms, assuming that the WSC content in different root components sampled first at the early growing stage in early April was the storage capacity, the higher nutrient contents indicated the larger storage capacity. As Fig 1 shows, the nutrient storage capacity of the root collars and 2-year old roots of the 1a and 2a plants was relatively large, whereas that of the root collars and 2-year old roots of the 3a plants was significantly reduced. Assuming that the change rate of the WSC contents in different root components at different growth stages was the physiological metabolic activity, the higher rate of change indicated the more intense physiological metabolic activity. As shown in Table 1, the physiological metabolic activity of different root components of the 1a plants at the resprouting stage was the most intense. For the period most intense physiological metabolic activity, for the period of the most intense physiological metabolic activity was partly lagged behind in of the 2year or 3-year roots of the 2a plants, and was generally lagged behind in different root components of the 3a plants. Both the reduced storage capacity and the lagged activity were important signs of aging. In the *A*. *scoparia* population, the 1a plants were juvenile, the 2a plants were young and the 3a plants were old. The aging signs which were indicated by the WSC contents and the change rates in different root components were consistent with the fact situation.

### Nutrient storage or activity of different root components and competitiveness of different individuals

New shoots from the root collars, as well as growth before leaf photosynthesis, require large consumption of the nutrients stored [31]. *A*. *scoparia* is a light-demanding plants [32], and nutrient storage capacity and activity of its root components have great impacts on the nutrient supply for aerial organs and the growth rate after resuming growth. In general, for the root components, especially the root collars, the greater the storage capacity, the greater the nutrient supply for the aerial organs. In early spring, the higher the activity, the earlier resprouting will be and the faster the aerial organ grows. The growth rate at the early growing stage determines the use of light resources throughout the whole growing season in the population or the community. Based on the nutrient storage capacity and activity of the root collars in the *A*. *scoparia* population, the 1a and 2a plants occupied the top positions in the population or the community, respectively, exhibiting strong competitive advantages. However, the competitiveness of the 3a plants was disappointing.

### Root collars survival time and maximum life span of *A*. *scoparia*

Dicotyledonous perennial herbaceous plants of the taproot type to maintain their perennial features mainly rely upon the resprouting or the vegetative propagation of the root collars year after year. Similar to the other apical dominance plants, *A*. *scoparia* individuals grow out more branches after removing the top growth within the growing season, such that only when the aboveground part gets old and dies at the end of the growth season appear development of the aerial parts on the root collars. Therefore, the *A*. *scoparia* root collar resprouting only forms a new generation every year. According to the generation computing for the happening resprouting on root collars, autumn-born seedlings can be overwintering with the rosette plant-type and their growth point of the root collars in the following year can continue to grow. So, the next spring, the root collars and the roots of the 1a plants (the lst generation) actually lived 2-year (two growing seasons). By contrast, the root collars and the taproots of the 2a plants (the 2nd generation) and the 3a plants (the 3rd generation) already lived through 3year and 4-year, respectively. In this way, the life spans of the root collars and the taproots can be evaluated from the age class of *A*. *scoparia* plants [43, 44]. In the present research, the survival time of the root collars of the 1a-3a sample plants was 2-year to 4-year old. Although 4a plants (root collars and taproots living through 5 years) were found in the *A*. *scoparia* population while sampling, their sample size was still insufficient. Therefore, the maximum life span of individuals of the *A*. *scoparia* population is 5 years in grasslands of northeast China.

## Conclusions

With regard to *Artemisia scoparia* in grasslands in northeast China, the nutrient storage capacity and the activity of the root collars and 2-year old roots of the 1a and 2a plants were larger, whereas that of the root collars of the 3a plants and all 3-year old roots was significantly decreased. During the period in which nutrients of *A*. *scoparia* root components were the most active in all root components of the 1a plants at the resprouting stage, partly lagged in the 2-year and 3-year old roots of the 2a plants, and generally lagged in all root components of the 3a plants. As a whole, the change rates in the WSC contents in *A*. *scoparia* root components of plants of three age classes were negative before mid-May, and the rates were generally relatively large. In the early stage of the growing season, all the individual storage nutrients and the new products of photosynthesis were used to the overground plant growth and organ development, so as to improve the competitive ability of the population in the community.

From the early resprouting stage in early April to the vigorously vegetative growth stage in late June, the transport of nutrients in the root components of the juvenile plants (1a) and old plants (3a) of the *A*. *scoparia* population regulated from logarithmic rule to linear rule with the growth process. In the root system of *A*. *scoparia*, the old root components (the 3-year roots) of the young plants (2a) demonstrated aging signs, whereas all the root components of the old plants (3a) already aged. There is finiteness on the resprouting, namely vegetative propagation generations of *A*. *scoparia* individual root collar, seed germination or sexual reproduction will be the main way to maintain the population.

## Supporting information

S1 Fig The mark of the picture on plant individuals of different age classes and the root components of *Artemisia scoparia* in early April. (PDF)
