# Supplementary material for: Water-soluble carbohydrates of root components and activity rhythms at vegetative growth stage of Artemisia scoparia in northeastern grassland of China
Source: PLoS One. 2017 May 9;12(5):e0176667. doi: 10.1371/journal.pone.0176667 (PMC5423605; doi:10.1371/journal.pone.0176667)
Supplement: S1 Fig — (PDF) [file pone.0176667.s001.pdf]

Supporting Information

Water-soluble Carbohydrates of Root Components and Activity Rhythms at Vegetative Growth Stage of *Artemisia scoparia* in Northeastern Grassland of China

Shiyu Wang, Yunfei Yang*, Heng Zhi

Key Laboratory of Vegetation Ecology, Ministry of Education, Institute of Grassland Science, Northeast Normal University, Changchun, Jilin province, P.R. China

* Tel.: +86 431 85099590; fax: +86 431 85693965.

E-mail address: yunfeiyangcncn@sina.com (YY).

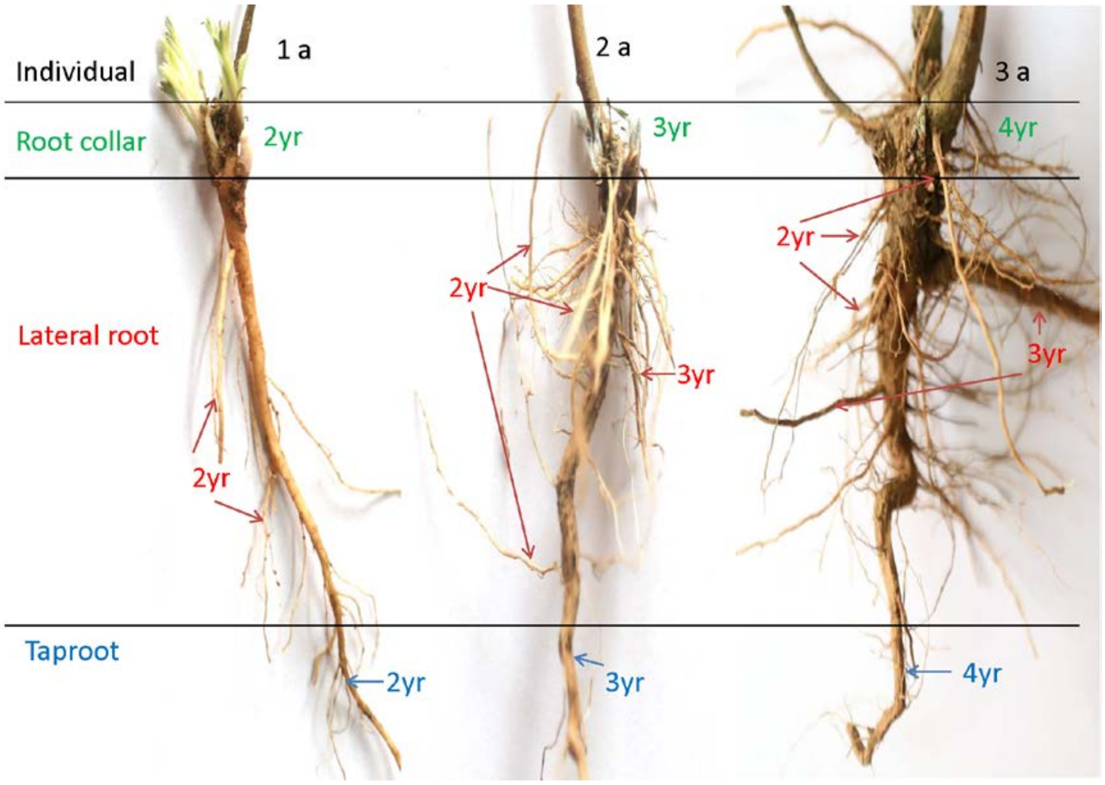

S1 Fig. The mark of the picture on plant individuals of different age classes and the root components of *Artemisia scoparia* in early April.

Notation: It set up four sections in the pictures to mark the age classes of individuals (a), years of life of the root collar/lateral root/taproot (yr), respectively. Need special explanation is that the age is times on the root collar resprouting (vegetative propagation) from generation to generation in the individual section. Among them, the toproot of 1a plant individuals and all 2-year old lateral roots are the flesh root, the taproots of 2a plant individuals and all 3-year old lateral roots have appeared different degree of lignification, the taproots of 3a plant individuals have obviously appeared lignification.
